# Method for Determining Regional Reference Values of Metal Content in Biological Substrates and Their Intake into the Body via Drinking Water

**DOI:** 10.3390/ijerph18189903

**Published:** 2021-09-20

**Authors:** Yulia Tunakova, Artur Shagidullin, Vsevolod Valiev, Svetlana Novikova, Rashat Faizullin

**Affiliations:** 1 Department of General Chemistry and Ecology, Kazan National Research Technical University named after A. N. Tupolev (KNRTU–KAI), 420126 Kazan, Russia; sweta72@bk.ru; 2 Research Institute for Problems of Ecology and Mineral Wealth Use of Tatarstan Academy of Sciences, 420087 Kazan, Russia; Artur.Shagidullin@tatar.ru (A.S.); podrost@mail.ru (V.V.); 3 Institute of Fundamental Medicine and Biology, Kazan Federal University, 420012 Kazan, Russia; RIFajzullin@kpfu.ru

**Keywords:** metals, drinking water, biosubstrates, reference values, neural network technologies, intake into the body

## Abstract

Natural and manmade flows of matter form complex metal associations in the body of residents living in certain territories, which leads to functional disorders in their bodies and the depletion of adaptive reserves. It is possible to assess the distribution of metals in the body only taking into account its biogeochemical localization. The question arises about the methodological approach to the determination of regional reference values of the concentrations of metals in biosubstrates of residents of different territories, to which this study was devoted. A designed and trained neural network was used, reflecting the relationship between the concentrations of metals in consumed drinking water and biosubstrates of the body, taking into account the physiological characteristics of the tested group of children and adolescents, based on the regional reference values obtained. Neural network regression methods allowed the calculation of nonlinear dependences of indicators of the state of the internal environment of an organism with external factors, and localized reference values determined in such calculations the indicators of the base state, being guided by the intensity of external factors, which should be assessed. The results of this study are intended for patient-oriented diagnosis and the treatment of eco-conditioned microelementosis in individual locations.

## 1. Introduction

The stability of the body’s elemental composition is one of the most important and mandatory conditions for its normal functioning. Each metal has its own safe range of concentrations in tissues, which allows the physiological state of the body and its biochemical functions to be adequately maintained. However, metals can be toxic if they accumulate in the body. There has been a sharp deterioration in the health of the population at the individual and population levels in areas of increased technogenic and toxic background. A present inhabitant of a large city is subject to chronic intoxication with doses of metals coming from various anthropogenic sources [1,2,3,4].

The territories of different regions differ significantly in their natural climatic, biogeochemical, and environmental characteristics. Therefore, the majority of residents in certain regions are characterized by the peculiarities of the metals content in the body. This is due to the specific metal intake and the biochemical characteristics of the area. Thus, the average metal content in the body of a resident in one region often differs from that in another region, while at the same time being within the safely level. Metals that enter the human body orally and with inhaled air are accumulated in its biosubstrates. Therefore, one of the most reliable methods that characterize the polymetallic impact on public health is the assessment of the metals content in biosubstrates. It is necessary to determine the safe ranges of metal concentrations in the organisms’ biosubstrates of each region’s inhabitants with regular updates. In addition, it is necessary at the same time to choose the correct control group of subjects and the study area [1,5,6,7].

The most informative or diagnostic biosubstrates are those that are involved in the processes of transport, excretion, and accumulation of metals [1,3,8]. In population-based studies, human hair (nails), blood (whole or serum), and urine (morning or daily) are usually used as such diagnostic biosubstrates. At the same time, hair reflects the total pool of metals in the body, blood—the intensity of their metabolism, and urine—the intensity of their elimination from the body. It is necessary to compare the content of metals in urine, blood, and hair, and evaluate their ratio [9,10,11].

Currently, there are no approved reference values of metals concentration in the biological substrates of inhabitants. Many laboratories analyze biosubstrates and set their own reference ranges that are not comparable. Reference values (RV) and reference intervals (RI) were defined by the International Federation of clinical chemistry (IFCC). Specific ranges are made for each gender and age group, based on geographical location. Based on the literature, it should be understood that the definition of reference intervals is a complex task and its solution is necessary for each large area of the territory. The results obtained should be constantly updated along with the modification of existing analytical tools. For many elements, the reference ranges of concentrations depend on the analytical method used for determination [5,11,12,13,14,15].

Uniform reference intervals for children of different ages are incorrect for assessing the metals content in diagnostic biosubstrates. They can lead to the incorrect diagnosis and classification of diseases [16,17,18,19,20,21,22]. Most studies of metal content in human biosubstrates have covered young children and adults, but the information on adolescents is extremely limited. We need a clear control group. To achieve this, it is advisable to choose an ecologically unfavorable territory, and make a representative sample of children on such a territory according to the values of biological, social, and hygienic criteria [6,9,17]. In regional surveys of biosubstrates’ composition, it is necessary to undertake research following uniform experimental conditions [16,18,19].

In this paper, we present the results of the analysis and statistical processing of carefully selected data research on the content (in different combinations) of 10 metals (Zn, Cu, Fe, Ni, Pb, Cd, Cr, Mn, Sr, and Mg) in line with various diagnostic biosusbtrates of healthy adolescents. The presented reference values were calculated according to the materials of research conducted at the National Center for the Protection of Family, Motherhood and Childhood, and later at the Department of Pediatrics of Kazan State Medical University and in the laboratory of biogeochemistry in the Research Institute for Problems of Ecology and Mineral Wealth Use of Tatarstan Academy of Sciences.

Part of the data were previously published [23,24,25,26], and some part are being published for the first time. These materials are the results of the population “background” study, regional standards for the content of metals in the adolescents’ biosubstrates living on the territory of certain biogeochemical provinces, covering the cities of Kazan, Naberezhnye Chelny, and Nizhnekamsk. The studied age group was 12–16 years old. They are all city dwellers and did not have any chronic diseases at the time of the study. The total sample size for blood serum was 55, for urine—78, and for hair—235.

The designed and trained neural network was used based on the obtained regional reference values. This network reflects the relationship between the concentrations of metals in drinking water and biosubstrates of the body, taking into account the physiological characteristics of the tested group of adolescents.

The localized assessment of metal concentrations’ reference ranges in the human body is intended for the development of personalized medicine methods for the prevention of pathologic conditions.

These data were based on narrow homogeneous samples of children and adolescents with similar living conditions. The pathologic conditions derive from microelementosis, their diagnosis, and treatment.

It is known that the relationships between the water–food intake of metals into the body by their retention with the formation of their individual microelement statuses are complex and, as a rule, nonlinear. Therefore, the methods of linear modeling cannot adequately describe the entire range of relationships between factors affecting the content of metals in the human body [4,7].

Neural networks, in comparison with traditional modeling methods, make it possible to operate with incomplete initial data, and they are also able to reflect nonlinear dependencies and select correction coefficients [25].

## 2. Materials and Methods

To determine a wide range of metals from biosubstrates, various methods are currently used: X-ray fluorescence analysis (XRF), neutron activation analysis (NAA), voltammetry, spectral methods—atomic absorption (AAS), atomic fluorescence (AFS), atomic emission spectroscopy (AES), including inductively coupled plasma (ICP AES), spectrometry and mass spectrometry, and inductively coupled plasma mass spectrometry (ICP-MS).

XRF can be successfully used for the determination of many metals, but the specific features of some samples impose restrictions on the accuracy and reproducibility of the analysis and require special standards of the same type for XRF [22].

The NAA method is a multi-element method. It can be used without reference samples. It has a high sensitivity, but is an expensive method, which limits its use for numerous determinations of the metal content in the biosubstrates [27].

Voltammetry can be used to detect heavy metals in biosubstrates, including hair. This method is affordable, quite cheap, but allows you to determine a limited number of metals at the same time and requires careful and time-consuming sample preparation [28].

In the analysis of biosubstrates, ICP AES and ICP MS methods complement each other: for the determination of macro-components, it is better to use atomic emission spectrometry, for the determination of micro components—mass spectrometry, but for numerous determinations of the metal content in samples, these methods are of limited use, due to the high cost [29].

The generally accepted method of the quantitative analysis of biosubstrates is atomic absorption spectrometry, as it is the most selective, reproducible, and relatively inexpensive method that allows the fast, high-quality, and sufficiently high sensitivity determination of trace element concentrations in various biosubstrates [27,29]. The flame atomizers allow the determination of Na, K, Rb, Cs, Ca, and Mg; for the determination of Pb, Zn, Cd, Cr, Cu, As, Se, Mn, Ni, Fe, etc., variants with nonflammable atomizers are usually used [30].

To obtain regional reference values, statistical approaches are traditionally used [18,31]. Modern assessments of the qualitative state of various environments used in population, biogeochemical, sanitary-hygienic, and medical research use threshold models. They are based on determining the degree or probability of exceeding certain control values (background, reference) by the parameters of the system under consideration. Therefore, the determination of such values is a critical issue, both as the assessment of different media in a wide sense and specific content-setting objectives of various substances [20,31,32].

Hair samples taken from the occipital part of the head along the entire length (proximal and distal sections together), blood serum (standard selected from the ulnar vein), and daily urine (without taking into account the volume of diuresis) were considered. Concentrations of trace elements were given in µg/g (hair) and µg/mL (blood serum and urine). Calibration solutions (basic and working) were prepared on the basis of state standard samples (1 g/L) by appropriate dilution with bidistilled water. The measurement of the concentrations of Zn, Cu, Fe, Sr, and Mg in the blood serum was carried out with a preliminary dilution of 1:2 with bidistilled water or in a TCA (trichloroacetic acid) filtrate (Cr, Pb, and Mn). To obtain the TCA filtrate, the hydrolysis of whey proteins with hydrochloric acid (reagent grade) was carried out. For this, 0.75 mL of 1.5% HCl solution was added to 1.5 mL of blood serum and incubated for 1 h at 37 °C. After hydrolysis of the proteins, they were precipitated with 0.75 mL of 20% TCA (trichloroacetic acid), the final dilution was 2 times, and after 1 h, they were centrifuged for 10 min at 1500 rpm. The supernatant liquid—TCA filtrate—was taken for analysis [33]. Urine samples (10 mL) were centrifuged at 1500 rpm, after which Zn, Sr, Cr, and Pb were determined in an aliquot directly, and Mg after dilution 100 times (0.1 mL was brought to 10 mL with bidistilled water). Hair samples were weighed and subjected to dry ashing in a muffle furnace at 450 °C, and the resulting ash was treated with 2 mL of concentrated HCl and then brought to 15 mL of 1 N of HNO_3_.

For the purposes of convergence and the uniform representation of concentration values, we used only data obtained by the atomic absorption method (AAS-3, SA10-MP, AAnalyst 400, AAnalyst 800 devices). The results of photocolorimetric, emission, X-ray fluorescence, mass-spectral, and other methods were not considered.

Due to the significant asymmetry of analytical samples (the normality of the distribution was checked by the Shapiro–Wilk test), medians and quartile ranges were used as their statistical characteristics. Accordingly, the limits of the standard intervals are the lower (25%) and upper (75%) quartiles. They are also offered by us as intervals of acceptable content (standards). The nonparametric Mann–Whitney U test was used as a paired method for evaluating the statistical significance of differences between two independent samples. In addition, a variation analysis was carried out and the limits of deviations of the final estimates were established. Statistical processing of the research results was carried out using a specialized software package STATISTICA V. 6.1 (StatSoft, Tulsa, OK, USA).

The total sample size was: blood serum n = 55, urine n = 78, and hair n = 235. The samples were preliminarily assessed for normal distribution by the Shapiro–Wilk W test. Due to the significant asymmetry of analytical samples, it was decided to abandon the use of mean values and, accordingly, their standard deviations, and consider the medians and quartile range of samples as statistical characteristics.

The characteristics of the equipment used in the study are as follows:

Atomic absorption spectrophotometer AAS-3, manufactured by VEB Carl Zeiss JENA (Germany); an optical two-beam system based on an Ebert mirror monochromator with two diffraction gratings; a photomultiplier tube and a deuterium nonselective absorption corrector; spectral range of 200–700 nm, and detection limits in an aliquot of 0.005–0.001 mg/L, depending on the metal being determined. The spectrophotometer uses atomic absorption spectral analysis using flame atomization. The flame type “acetylene-air” was used for the analysis [34] to assess the content of metals in hair.

The SA-10MP spectrometer (manufactured by the Kazan Optical and Mechanical Plant, Russia) is a single-beam spectroanalytical instrument with a background corrector, flame and tungsten spiral atomizers. It has a spectral range of 190–900 nm, detection limits with a flame atomizer of 10^−4^–10^−5^ mg/L, and a tungsten spiral of 10^−5^–10^−7^ mg/L [35]. It was used to assess the content of metals in hair, serum, and urine.

Spectrophotometers AAnalyst 400 and AAnalyst 800 manufactured by Perkin Elmer (USA) are different modifications of atomic absorption spectroscopy with both flame and electrothermal atomization. Its spectral range is 190–870 nm, taking into account the nonselective absorption of the Zeeman and Quad Line. They provide the ability to determine most metals with a detection limit of 0.005–0.0001 mg/L [36]. They were used to assess the metal content in hair and urine.

Zn was determined with a detection limit of 0.001 μg/mL, Cu 0.001 μg/mL, Fe –0.01 μg/mL, Cr 0.005 μg/mL, Cd 0.0008 μg/mL, Mg 0.001 μg/mL, Sr 0.003 μg/mL, Ni 0.006 μg/mL, Pb 0.005 μg/mL, and Mn 0.002 μg/mL.

It is possible to simulate the characteristics of the intake of metals in the body using the reference ranges obtained in this study for the content of various metals in biological substrates. As a paradigm of the model, MLP-type neural networks were chosen, the structure of which is determined empirically and is determined by the potential complexity of the data being processed. A network reduction method based on multicriteria optimization was selected as a method for training neural networks [25].

## 3. Results

Table 1, Table 2 and Table 3 present the summary data obtained for the general selection of all children. However, it should be noted that the content of some metals in different cities in the hair differed significantly. These cases are noted in the note. There were no statistically significant differences in the content of metals in blood serum and urine in children from different cities.

As a result of the comparison of different data series, it was found that in the hair of children aged 12–16 years living in the Republic of Tatarstan, the content of essential metals in the norm should be: Zn = 120.5 µg/g; Cu = 9.0 µg/g; Mg = 22.7 µg/g; Fe = 16.6 µg/g. The content of toxic metals in hair should normally be: Cd ≤ 0.25 µg/g; Pb ≤ 4.13 µg/g; Sr ≤ 14.0 µg/g; Cr ≤ 0.8 µg/g. (Table 1).

The concentration of essential metals in the blood serum should not be lower than Mg = 18.4 µg/mL, Zn = 0.759 µg/mL, Fe = 1.21 µg/mL, and Cu = 0.766 µg/mL. At the same time, the concentration of toxic metals in the blood serum should be: Cr ≤ 0.039 µg/mL; Sr ≤ 0.12 µg/mL; Pb ≤ 0.034 µg/mL (Table 2).

The excretion of metals in the urine also has its own thresholds. In this case, only high concentrations of metals are an unfavorable sign, indicating either a violation of kidney function, or an increased intake of the body from the environment. For example, the values of concentrations in the urine are: Zn ≤ 0.45 µg/mL; Pb ≤ 0.110 µg/mL; and Mg ≤ 100 µg/mL (Table 3).

## 4. Discussion

The estimation of the variational volatility of the data showed a significant spread of metal concentrations, even within the same type of samples. Especially high values of variational volatility were achieved in daily urine samples, regardless of the year and the laboratory that provided the test results. This fact is also reflected in the quartile range represented by the lower and upper thresholds of the reference values.

Table 4 shows the values of variations for different metals for all the mediums studied. In the hair of adolescents, the greatest variability in Mg and Sr (58%) content was observed and the lowest for Zn (11%).

In daily urine samples, Pb (77%) concentrations showed the highest variability, and Mg (36%) concentrations showed the lowest variability.

The data on the concentration of metals in blood serum were most stable. The greatest variability was observed in the content of Fe (24%), the lowest in Mg (11%). Thus, according to the degree of variability of the metal content in general, biosubstrates were arranged in a descending series: urine-hair-blood. One exception is noted here: alkaline-earth metals such as magnesium and strontium showed the maximum variability in the content not in the urine, but in the hair of children.

This fact should be taken into account when developing regional standards, as well as when comparing data series obtained from different sources.

As a result, localized reference limits of the metal content in biosubstrates of children and adolescents, who live in the territory of the Republic of Tatarstan, were obtained. We proposed the use of the calculated reference concentrations of metals as regional thresholds for the content of essential and toxic metals in different biosubstrates.

The designed hybrid smart model [25] was used to estimate the intake of metals with drinking water. The model consists of two cascade-connected neural networks covering the concentrations of metals in biosubstrates and in drinking water, taking into account the anthropophysical characteristics of the subject’s body.

It is possible to reasonably adjust the norms of daily requirement for microelements using the nonnetwork model developed by us for assessing retention [25] and the reference values obtained in this study. In turn, this will make it possible to determine the degree of the adolescents’ body supply with microelements.

For this task, the obtained values of the regional reference values of the concentrations of metals in biological substrates were fed to the input of the developed neural network, which calculates the corresponding level of intake.

The intake of metals into the body with drinking water was estimated based on the obtained ranges of regional reference values of metal concentrations in biosubstrates (Table 5).

## 5. Conclusions

We have previously shown that the content of metals in biosubstrates is interrelated with their intake with drinking water through such an indicator as retention. We built models of the relationship between indicators of the state of the internal environment of the body with external factors, using neural network algorithms that allow us to evaluate nonlinear dependencies, while localized reference values represented the basic state of the internal environment, based on which it is possible to assess the intensity of external factors, in this case, the permissible concentration of metals in drinking water.

We proposed an approach for assessing the intake of metals into the body based on their content in biosubstrates, as the daily requirements for essential metals are indicated without taking into account regional population characteristics of supply.

Our proposed method for assessing the intake of metals in the body based on their content in biosubstrates and taking into account the retention of metals in the body using a cascade hybrid intelligent system does not require expensive laboratory studies and makes it possible to quantitatively determine the balance of metals in the body. The simplified structure of the neural network regression model (reducing the number of inputs) provides sufficient accuracy, and the reduction in neural networks increases the adequacy of the models.

The calculated reference intervals of the content of metals in biosubstrates and their intake with the consumed drinking water can be used as a tool for clinical diagnostics and the development of adequate methods for correcting the revealed imbalance, both at the individual and population levels.

The determination of the metal content in diagnostic biosubstrates is an indicator of the biogeochemical and ecological characteristics of the territory. The calculated reference intervals of metal content in biosubstrates can be used as a tool for clinical diagnosis and the development of recommendations for the optimal intake of trace elements in children suffering from microelementosis.

## Figures and Tables

**Table 1:** Variational indicators of metal content in hair (µg/g) *.

Metal	Median	Lower Quartile (Lower Threshold)	Upper Quartile (Upper Threshold)
Zn	135.0	120.5	155.2
Cd	0.163	0.092	0.258
Cu	11.3	9.0	15.0
Mn	0.356	0.240	0.510
Ni	0.128	0.100	0.210
Pb	2.995	1.680	4.130
Cr	0.510	0.385	0.805
Sr	8.65	3.66	14.0
Mg	55.2	22.7	80.8
Fe	30.0	16.6	55.5

* Note: The content of some metals in the hair of children in different cities was statistically significantly different (*p* < 0.05): in Nizhnekamsk: Cd M = 0.44 (0.2–0.72), Ni M = 0.52 (0.28–0.73), Zn M = 115.5 (93.6–122.7); in Naberezhnye Chelny: Ni M = 0.65 (0.40–1.0), Mn M = 1.85 (0.66–4.0).

**Table 2:** Variational indicators of metal content in blood serum (µg/mL).

Metal	Median	Lower Quartile (Lower Threshold)	Upper Quartile (Upper Threshold)
Mg	20.7	18.4	24.5
Zn	0.908	0.759	1.255
Cr	0.031	0.026	0.039
Fe	1.604	1.210	2.220
Sr	0.076	0.066	0.120
Cu	0.955	0.766	1.522
Pb	0.030	0.025	0.034

**Table 3:** Variational indicators of metal content urine (µg/mL).

Metal	Median	Lower Quartile (Lower Threshold)	Upper Quartile (Upper Threshold)
Zn	0.330	0.100	0.450
Cr	0.015	0.005	0.030
Pb	0.035	0.008	0.110
Sr	0.100	0.046	0.250
Fe	0.200	0.053	0.350
Mg	60.3	38.6	100.4

**Table 4:** The variational volatility of observation series of the metal’s concentration in different biological substrates, %.

Metal	Hair	Urine	Blood
Cd	43.5	-	-
Cr	24.5	66.6	16.1
Cu	20.3	-	19.7
Fe	44.6	73.5	24.5
Mg	58.8	35.9	11.1
Mn	32.5	-	-
Ni	21.8	-	-
Pb	43.9	77.1	16.6
Sr	57.6	54.0	13.1
Zn	10.7	69.6	16.4

**Table 5:** Concentration range of metals entering the body with drinking water consumption (µg/L).

Metal	Concentration of Metals in Drinking Water
Zn	0.019–0.033
Cr	0.00078–0.002
Fe	0.074–0.1
Sr	0.05–0.11
Cu	0.0013–0.0021
Pb	0.001–0.007
